# Health professionals for the future

**DOI:** 10.1186/1472-6963-14-S2-P43

**Published:** 2014-07-07

**Authors:** Armin Gemperli, Beat Sottas, Gerold Stucki

**Affiliations:** 1 Department of Health Sciences and Health Policy, University of Lucerne, Lucerne, Switzerland; 2 Swiss Paraplegic Research, Nottwil, Switzerland; 3 Careum Foundation, Zurich, Switzerland

## 

Recommendations for an intersectoral policy for the education of health professionals have recently been stated in the Lancet Report “Health professionals for a new century” and by the WHO policy framework “Health 2020”. These postulates were further developed into an educational strategy by the Careum Foundation. In this framework four mutually dependent perspectives were defined: functions related to the population, to patients, to organisational development, and to increasing knowledge. It is stressed that besides the historically strong education for patient-related functions, the three other functions must receive the same attention with regard to regulation and financing. Hence, education for health professionals must lead to cross-functional and intersectoral thinking and makes cooperation skills a priority, besides promoting technical expertise. These skills require new approaches in methodology and didactics.

The Department of Health Sciences and Health Policy at the University of Lucerne, Switzerland, recently developed a Master’s program that complements clinical education with an education that aims at the other three functions. The program offers a case in point on how the current dialogue on the intersectoral policy for the education of health professionals can be put into practice.

The program approaches the complexity of health issues by examining them from an interdisciplinary perspective. Teaching modules are instructed by a diverse faculty of researchers with background in areas such as medicine, natural sciences, economics, law and the humanities. Besides the teaching of technical expertise, knowledge of health systems and services is broadly included in the curriculum. After the first semester, students can choose their major in the area of health and social behavior, health economics and health policy, health communication, research methods, human functioning sciences or health services research. The faculty works in close connection with local partners from academic institutions, industry and government. Together with these partners, research internships are offered to all students, which allow students to participate in a team outside University.

Conclusions so far are that students are highly motivated to grasp the complexity of health issues from an interdisciplinary perspective. Many students who were initially attracted by specific majors were introduced to a broader health perspective and gained insight in novel areas they would not have otherwise. The entire program is taught in English which attracts a broad international student body with diverse backgrounds and fosters cooperation skills and the cross-area dialogue during interactive sessions.

